# Factors affecting skilled delivery service utilization: a mixed-methods study in Ghana's West Akim Municipality in the Eastern Region

**DOI:** 10.3389/fgwh.2026.1795305

**Published:** 2026-06-17

**Authors:** Elizabeth Mensah, Emmanuel Kwasi Afriyie, Samuel Egyakwa Ankomah, Emmanuel Kumah, Nyarko Perpertual Nsiah, Precious Wonder Adekore, Godfred Otchere, Samuel Kofi Agyei, Adam Fusheini

**Affiliations:** 1 Department of Public Health, Presbyterian University, Asante Akyem Campus, Agogo, Ghana; 2 Emergency Medicine Directorate, Komfo Anokye Teaching Hospital, Kumasi, Ghana; 3 Department of Management, University of Cape Coast, Cape Coast, Ghana; 4 Department of Health Administration and Education, Faculty of Health, Allied Science and Home Economics Education, University of Education, Winneba, Ghana; 5 Department of Public Health, University of Otago, Dunedin, New Zealand; 6 Department of Healthcare Management, College of Business, University of Doha for Science and Technology, Doha, Qatar

**Keywords:** Ghana, health services utilization, maternal health, mixed-methods, skilled delivery

## Abstract

**Background:**

Maternal mortality remains a critical public health challenge in Ghana. Despite the country's ‘free’ Maternal Healthcare Policy, skilled delivery service utilization remains suboptimal, particularly in rural and semi-urban areas such as the West Akim Municipality. This study aimed to identify and contextualize the factors influencing the utilization of skilled delivery services in the West Akim municipality, with the goal of generating evidence to inform targeted interventions and health system strengthening.

**Methods:**

A convergent parallel mixed-methods, cross-sectional study was conducted in January 2025. The quantitative component surveyed 402 women aged 15–49 who had a live birth in the preceding year, using a structured questionnaire. Multivariable logistic regression was conducted to identify factors associated with skilled delivery service utilization. Concurrently, qualitative data were collected through 4 focus group discussions (*n* = 28), 20 in-depth interviews, and 16 key informant interviews with healthcare workers and community stakeholders. The data were analyzed using inductive thematic analysis.

**Results:**

The skilled delivery utilization rate was 75.4%. Key quantitative predictors included higher household income (AOR=2.8, 95% CI: 1.3–5.9), health insurance coverage (AOR=3.2, 95% CI: 1.8–5.7), and secondary/tertiary education (AOR=2.4, 95% CI: 1.2–4.6). Factors reducing utilization were husband-dominated decision-making (AOR=0.5, 95% CI: 0.3–0.9), living ≥5 km from a facility (AOR=0.4, 95% CI: 0.2–0.7), and perceived poor staff attitudes (AOR=0.4, 95% CI: 0.2–0.7). Qualitative findings revealed persistent indirect costs despite insurance, strong cultural trust in traditional birth attendants, prohibitive transportation barriers, and experiences of receipt of disrespectful care as key contextual barriers.

**Conclusion:**

Skilled delivery services utilization is influenced by a complex intersection of socio-economic, cultural, and health system factors. To improve coverage, policies must move beyond removing formal fees to addressing hidden costs, engage men and traditional providers, improve geographical access, and mandatorily promote respectful maternity care to rebuild community trust in the health system.

## Introduction

Maternal mortality remains a persistent global health challenge, disproportionately affecting low- and middle-income countries (LMICs) (1). In Ghana, the Maternal Mortality Ratio (MMR) is estimated at 308 deaths per 100,000 live births, a figure that remains alarmingly high and far exceeds the Sustainable Development Goal (SDG) target 3.1 of less than 70 (2, 3). While this is notably lower than the Sub-Saharan African average MMR of 536 (4), it remains far above the SDG target 3.1 of less than 70. Furthermore, skilled birth attendance in Ghana, though higher than the regional average of 64.3%, remains below the national target of 84%. Skilled delivery, defined as childbirth assisted by a trained professional, is a keystone intervention for preventing deaths from major obstetric complications (5, 6). However, its uptake in Ghana remains suboptimal and uneven, with significant rural-urban and socioeconomic disparities persisting (7, 8).

To address this, Ghana implemented the Free Maternal Healthcare Policy under the National Health Insurance Scheme (NHIS). The primary objective of this policy is to eliminate out-of-pocket payments for maternal health services and thereby increase access to and utilization of facility-based skilled delivery. Despite this concerted policy effort, a significant gap persists between this objective of financial risk removal and the actual utilization of skilled delivery services. This gap suggests that persistent non-financial and context-specific barriers undermine these initiatives. Literature reveals a multi-layered web of determinants behind this utilization gap. These include remaining costs associated with insurance schemes (9, 10), health system challenges such as geographical inaccessibility, poor infrastructure, and perceptions of disrespectful care (11, 12), and socio-cultural factors such as gender-based decision-making, norms privileging home birth, and trust in traditional birth attendants (13). These factors intersect to shape a woman's choice of care delivery.

While the challenge is well documented at the national level, effective intervention requires granular, district-level evidence. In Ghana's Eastern Region, the West Akim Municipality exemplifies a setting where multiple barriers likely converge, as utilization of skilled delivery services remains below expectations: in 2023, only 63.1% of expected deliveries occurred under skilled care, far below the national target of 85% (7).

This study, therefore, aimed to explore factors influencing utilization of skilled delivery service among women of reproductive age in this municipality. Using a mixed-methods approach, it sought to quantify key associations and qualitatively explore lived experiences and contextual narratives by: 1) identifying socio-economic factors; 2) examining cultural beliefs and practices; and 3) assessing health system factors related to accessibility and availability. The findings are intended to generate actionable, context-specific evidence to inform local health planning. This study contributes to the broader discourse on achieving equitable maternal health coverage in LMICs.

## Methods

### Study design and setting

This study employed a convergent parallel mixed-methods design within a cross-sectional framework. As defined by Creswell & Clark, this design involves a concurrent but separate collection and analysis of quantitative and qualitative data, with the results integrated to provide a complete understanding of the research problem (14). Quantitative and qualitative data were collected concurrently in January 2025. The two datasets were analyzed separately and then merged during the interpretation phase. This integration allowed for triangulation, where findings from each method were compared and synthesized to develop a comprehensive and validated understanding of the determinants of skilled delivery utilization (15).

The study was conducted in the Asamankese District within the West Akim Municipality of Ghana's Eastern Region (16). The district was purposively selected as a study setting based on specific criteria: its central location, mixed urban-rural characteristics, administrative relevance as a municipal capital, and accessibility. A primary motivator for the study was the district's documented suboptimal skilled delivery coverage as the data from the Ministry of Health, Ghana. These features, in line with Patton's assertion, made it a suitable microcosm of the broader Eastern Region, which allowed for a broader examination of factors influencing utilization of skilled delivery services in a context representative of regional diversity (17).

### Study population and sampling

**Figure 1:**
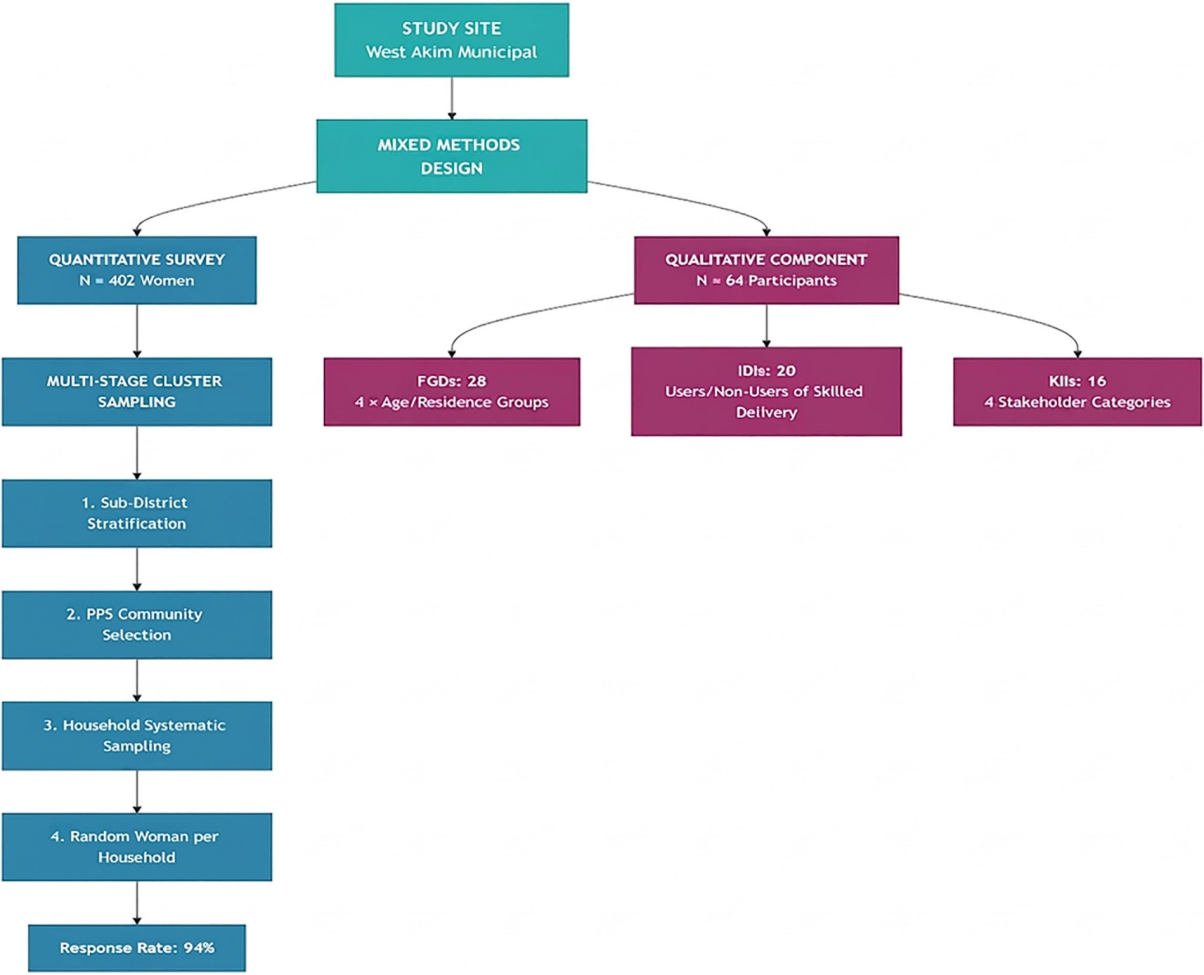
Sampling strategy and participant flow diagram.

The target population was women aged 15–49 years who had a live birth in the 12 months preceding the survey and were residents of the municipality for at least six months. Women who were critically ill or unable to provide consent were excluded. The sample size for the quantitative component was calculated using the Cochran's single population proportion formula (18). Assuming a 50% prevalence of skilled delivery (*p* = 0.5), a 95% confidence level (Z = 1.96), and a 5% margin of error (d = 0.05), a minimum sample of 385 was derived. Accounting for a 10% non-response rate, the final sample was 428. A multi-stage cluster sampling technique was used, a common and efficient approach for population-based surveys in low-resource settings (19) as outlined in Figure 1: 1) stratification of the municipality into sub-districts; 2) probability proportional to size (PPS) selection of communities (clusters); 3) systematic random sampling of households within selected clusters; and 4) random selection of one eligible woman per household. In total, 402 women completed the survey (response rate: 94%).

Participants for the qualitative component were selected using purposive sampling to capture diverse perspectives and experiences until thematic saturation was achieved (20). Saturation was determined as the point where no new substantive codes or themes emerged from the last two consecutive IDIs and FGDs within a participant category. This included a four Focus Group Discussion (FGD) (*N* = 28 total), stratified by age (<25 vs. ≥ 25 years) and residence (rural vs. urban/peri-urban) to explore group norms within sociodemographic segments. There were in-depth interviews (IDIs) with twenty women, selected to include both users and non-users of skilled delivery, to understand individual decision-making pathways. There were also Key informant interviews (KIIs) involving sixteen stakeholders of four healthcare workers, four traditional birth attendants (TBAs), four religious’ leaders, and four community/traditional leaders. These informants provided insights into the health system, socio-cultural norms, and community governance influencing maternal health choices (21).

### Data collection

A structured questionnaire was developed to capture data across five key domains (1): socio-demographic characteristics; (2) obstetric history; (3) economic factors (insurance status, costs incurred, employment); (4) socio-cultural dimensions (household decision-making, birth preferences, community norms); and (5) health system factors (distance to facilities, travel time, transport availability, perceived facility readiness, and staff attitudes). The study was pre-tested on 20 women in a non-sampled community to assess clarity, flow, and timing. Minor revisions to wording and question sequence were made based on feedback to ensure content validity and local relevance. Final data collection was administered electronically by trained research assistants using the Kobo Toolbox platform on mobile devices, a method shown to improve data quality and efficiency in field research (22). The survey took approximately 25–35 min to complete.

Qualitative data were gathered through semi-structured guides for FGDs, IDIs, and KIIs. The guides explored themes parallel to the quantitative domains but were designed to allow for in-depth probing and narrative elaboration (17). All sessions were conducted in the local language (Twi) by trained facilitators to ensure participants could express nuanced cultural and emotional experiences freely. Translation followed a two-step verification protocol: 1) verbatim transcription in Twi by a native speaker; 2) forward translation to English by a bilingual research assistant; and 3) back-translation of a random 10% sample of quotes by an independent linguist to verify semantic equivalence. With participant consent, discussions were audio-recorded, later transcribed verbatim, and translated into English to preserve linguistic and contextual nuance. Each FGD, facilitated by a moderator and a note-taker, lasted 45–60 min, while individual interviews (IDIs and KIIs) ranged from 30 to 60 min.

### Data analysis

Data preparation and management were performed using Microsoft Excel (Microsoft Corporation, Redmond, WA, USA). Statistical analyses were conducted with STATA version 17 (StataCorp LLC, College Station, TX, USA). Descriptive statistics (frequencies, percentages, means, and standard deviations) summarized participant characteristics and key variables. Bivariate analyses (Chi-square tests) assessed associations between independent variables (socio-economic, cultural, health system factors) and the primary outcome (skilled delivery utilization: Yes/No). Variables significant at *p* < 0.10 in bivariate analysis were entered into a multivariable logistic regression model to identify independent predictors, controlling for confounders. A *p*-value threshold of <0.10 was selected for inclusion (rather than <0.05) to minimize the risk of residual confounding by excluding variables that might demonstrate significant adjusted effects despite modest unadjusted associations. Adjusted odds ratios (AORs) with 95% confidence intervals (CIs) and *p*-values <0.05 were considered statistically significant.

Additionally, a thematic analysis was conducted following Braun and Clarke's six-phase framework (23), a widely used method for identifying, analyzing, and reporting patterns within qualitative data. The qualitative component was conducted in accordance with the Standards for Reporting Qualitative Research (SRQR) (24), to ensure comprehensive and transparent reporting (see Supplementary Material: Table 1). Analysis began with repeated reading of transcripts for familiarization. An inductive approach was primarily used to generate codes from the data, which were then organized into broader themes that ultimately aligned with and expanded upon the study's original conceptual domains using MAXQDA software (version 20). To ensure rigor and minimize researcher bias, the research team engaged in regular peer debriefing sessions where codes and emerging themes were discussed and challenged. Coding disagreements were resolved through iterative consensus, reviewing the full transcript context to ensure codes reflected participant-intended meaning rather than researcher assumptions. Additionally, two coders maintained a reflexive journal to document and bracket preconceptions regarding traditional birth practices and healthcare access. Recurring patterns, illustrative quotes, and divergent views were identified to build a narrative explaining the quantitative findings. Triangulation of findings enhanced validity, with qualitative narratives providing context and depth to the statistical associations.

### Ethical considerations

Ethical approval was obtained from the Faculty of Health and Medical Sciences, Presbyterian University, Ghana (Ref No. PUG/ST/AS/24070035). All methods were in accordance with the Declaration of Helsinki. Permission was also secured from the West Akim Municipal Health Directorate. Written informed consent was obtained from all participants; for non-literate participants, the form was read aloud, and a thumbprint was obtained in the presence of a witness. For minors (15–17 years), assent and parental/guardian consent were secured. Confidentiality was maintained through anonymization of data, use of identification codes, and secure storage of electronic and physical records. Participation was voluntary, with the right to withdraw at any time without penalty. The study posed minimal risk, and interviews were conducted in private settings to ensure participant comfort.

## Results

The study integrated quantitative data with qualitative insights. The study findings are organized by the three study objectives: socio-economic factors, socio-cultural beliefs and practices, and health system factors affecting skilled delivery service utilization.

### Socio-demographic characteristics of participants

Of the 402 women surveyed, the majority were aged 25–34 (48.8%), married or cohabiting (74.1%), and had attained a secondary education (40.3%). More than half (50.7%) had two to three children, and 60.7% were employed (see Supplementary Material: Tables 2 and 3). In accordance with ethical protocols, adolescents aged 15−17 years comprised a small proportion of the sample (*n* = 15), and all provided both parental consent and personal assent prior to participation. The qualitative component of the study, which involved 64 participants, reflected a similar demographic profile, encompassing a range from young first-time mothers to older multiparous women.

Qualitative data were generated through three complementary methods: 28 postpartum women (within 12 months of delivery) participated in FGDs, and 20 postpartum women, including both skilled delivery users and non-users, took part in IDIs. To incorporate broader community and health system perspectives, 16 KIIs were conducted with healthcare workers, Traditional Birth Attendants, and local religious and community leaders. This mixed-methods approach allowed for a triangulated understanding of the factors influencing maternal health service utilization.

#### Theme 1: socio-economic factors and skilled delivery utilization: the interplay of financial means, insurance, and hidden costs

Economic factors were strongly associated with the use of skilled delivery services. Women from high-income households were significantly more likely to utilize skilled delivery (89.6%) compared to those from low-income households (62.7%, *p* < 0.001). Similarly, employed women and those with active health insurance coverage had significantly higher utilization rates (Table 1).

**Table 1:** Association between socio-economic factors and skilled delivery utilization.

Economic factor	Category	Skilled delivery (*n*, %)	Non-skilled delivery (*n*, %)	*p*-value
Household income	Low	84 (62.7)	50 (37.3)	<0.001
	Medium	132 (79.0)	35 (21.0)	
	High	86 (89.6)	10 (10.4)	
Employment status	Employed	212 (86.9)	32 (13.1)	<0.001
	Unemployed	90 (57.0)	68 (43.0)	
Health insurance	Yes	198 (88.0)	27 (12.0)	<0.001
	No	104 (60.5)	68 (39.5)	
Transport cost (GHS)*	Mean (SD)	12.4 (6.8)	18.7 (7.2)	<0.001

* GHS: Ghana cedis. Exchange rate at time of study (January 2025): 1 USD ≈ 15.0 GHS. Mean transport cost was approximately 0.83USD (skilled group) vs. 0.83USD (skilled group) vs. 1.25 USD (non-skilled group). Household income categories were defined by sample tertiles: low (<800 GHS/month), medium (800–1,500 GHS/month), high (>1,500 GHS/month).

While quantitatively a strong facilitator, qualitative narratives revealed significant limitations to this financial protection. Qualitative data revealed that even women enrolled in the NHIS faced financial barriers. Hidden costs for gloves, medicines, and supplies were frequently cited, alongside significant transportation expenses.

“Even though delivery is supposed to be free under the insurance, they still ask you to buy gloves and medicine. If you don't have cash at that moment, you are left with no choice but to go back home.” (FGD participant, rural, age 28)

Transport costs were particularly prohibitive for women in remote areas, especially during emergencies at night. A community leader noted:

“The biggest problem is money for transport. When labor starts at night, there are no cars, and if you find one, the fare is triple. Many families simply cannot afford it.” (KII, Community Leader)

#### Theme 2: socio-cultural beliefs and practices: cultural norms, household dynamics, and trust in alternative care

Socio-cultural norms significantly influenced delivery choices. Nearly one-third of participants (30.3%) agreed that childbirth is a natural process not requiring hospital care. Husband-dominated decision-making was reported by 36.3% of women, and 24.4% expressed a preference for TBAs or home delivery (Table 2).

**Table 2:** Socio-cultural beliefs and their association with skilled delivery.

Belief/practice	Agree *n* (%)	Neutral *n* (%)	Disagree *n* (%)	*χ*^2^, *p*-value
Childbirth is natural, no need for hospital	122 (30.3)	48 (11.9)	232 (57.7)	χ^2^ = 32.6, *p* < 0.001
Husband decides place of delivery	146 (36.3)	62 (15.4)	194 (48.3)	χ^2^ = 28.4, *p* < 0.001
Preference for TBA/home delivery	98 (24.4)	56 (13.9)	248 (61.7)	χ^2^ = 21.7, *p* < 0.001
Facility delivery linked to shame/stigma	74 (18.4)	68 (16.9)	260 (64.7)	χ^2^ = 11.5, *p* = 0.003

Qualitative narratives provided depth, revealing that trust in TBAs was rooted in cultural familiarity, emotional support, and shared belief systems. A TBA explained:

“They trust me because I am from here. I speak their language, I pray with them, and I stay with them throughout. At the hospital, you are alone with strangers.” (KII, TBA)

Religious beliefs also played a role, with some participants viewing successful delivery as primarily dependent on divine intervention. A religious leader shared:

“Some believe that if God has blessed the pregnancy, He will see the delivery through. Going to the hospital is sometimes seen as a lack of faith.” (KII, Religious Leader)

Male authority was a recurring theme. A young mother explained:

“My husband and his mother decided I should deliver at home with the TBA. He controls the money, and he said the hospital is too expensive and the nurses are rude.” (IDI, age 24)

#### Theme 3: health system accessibility and availability: geographical barriers, logistics, and experiences of care

Logistical and systemic barriers were critical deterrents. Women living within 5 km of a facility had significantly higher skilled delivery utilization (84.5%) than those living 5 km or more away (59.2%). Travel time exceeding 30 min, lack of transport (especially at night), and negative staff attitudes were all strongly associated with non-utilization (Table 3).

**Table 3:** Health system factors and skilled delivery utilization.

Health system factor	Category	Skilled delivery (*n*, %)	Non-skilled delivery (*n*, %)	*p*-value
Distance to facility	<5 km	196 (84.5)	36 (15.5)	<0.001
	≥5 km	106 (59.2)	73 (40.8)	
Travel time	<30 min	178 (85.2)	31 (14.8)	<0.001
	≥30 min	124 (62.9)	73 (37.1)	
Night transport available	Yes	218 (84.5)	40 (15.5)	<0.001
	No	84 (55.3)	68 (44.7)	
Staff attitude perceived as good	Yes	228 (83.5)	45 (16.5)	<0.001
	No	74 (55.2)	60 (44.8)	

This statistical association was powerfully illustrated by women's lived experiences of care. Poor road infrastructure and unreliable emergency transport were highlighted as major concerns. A woman from a remote community explained:

“During the rainy season, the road becomes a river. I went into labor during a storm, and the motorbike could not pass. We had to wait until morning, and I delivered on the way.” (IDI, age 32)

Staff attitudes profoundly influenced trust. Negative past experiences with healthcare workers deterred facility use. A healthcare worker acknowledged this challenge:

“We are overworked and sometimes frustrated, but I know some nurses shout at the women. When they go back to the village, they tell others, and then everyone is afraid to come.” (KII, Midwife)

### Multivariable logistic regression analysis

After adjusting for potential confounders, several factors remained independent predictors of skilled delivery utilization (Table 4). While parity (≥4) was included in the model due to its potential confounding effect, it did not remain a statistically significant predictor in the final adjusted analysis (*p* = 0.184). Higher household income (AOR=2.8, 95% CI: 1.3–5.9), employment (AOR=2.1, 95% CI: 1.2–3.8), and health insurance (AOR=3.2, 95% CI: 1.8–5.7) significantly increased the odds. Conversely, husband-dominated decision-making (AOR=0.5, 95% CI: 0.3–0.9), preference for TBAs (AOR=0.6, 95% CI: 0.3–0.9), distance ≥5 km (AOR=0.4, 95% CI: 0.2–0.7), and poor staff attitudes (AOR=0.4, 95% CI: 0.2–0.7) significantly reduced the odds.

**Table 4:** Multivariable logistic regression of factors associated with skilled delivery service utilization.

Variable	Adjusted odds ratio (AOR)	95% Confidence interval	*p*-value
Household income (high vs. low)	2.8	1.3–5.9	0.006
Employment (employed vs. unemployed)	2.1	1.2–3.8	0.009
Health insurance (yes vs. no)	3.2	1.8–5.7	<0.001
Husband decides place of delivery (yes vs. no)	0.5	0.3–0.9	0.018
Preference for TBA (yes vs. no)	0.6	0.3–0.9	0.021
Distance ≥5 km (vs. < 5 km)	0.4	0.2–0.7	0.002
Travel Time ≥30 min (vs. < 30 min)	0.5	0.3–0.9	0.014
Staff attitude (poor vs. good)	0.4	0.2–0.7	0.003
Education (sec/tertiary vs. none)	2.4	1.2–4.6	0.011
Parity ≥4 (vs. 1)	0.7	0.4–1.2	0.184

Adjusted for age, marital status, and parity. Parity ≥4 was included as a control variable but did not reach statistical significance in the final adjusted model.

## Discussion

The present study examined the multifaceted factors influencing skilled delivery service utilization among women of reproductive age in the West Akim Municipality of Ghana using a convergent mixed-methods approach. By integrating quantitative and qualitative evidence, the study provides a comprehensive understanding of how socio-economic, socio-cultural, and health system factors interact to shape women's delivery choices. Overall, the findings demonstrate that skilled delivery utilization is driven by a complex interplay of enabling and constraining factors, operating simultaneously at the individual, household, community, and health system levels. These results are consistent with broader evidence from LMICs and underscore the importance of context-specific, multidimensional interventions.

Socio-economic conditions emerged as a critical foundation shaping access to skilled delivery services. Quantitative analyses showed strong positive associations between skilled delivery utilization and higher household income, employment status, and health insurance coverage, confirming that economic empowerment substantially enhances women's ability to seek facility-based care. These findings align with existing literature from sub-Saharan Africa identifying poverty and financial constraints as persistent barriers to maternal healthcare utilization (25–27). The strong protective effect of health insurance (AOR = 3.2) reflects the intended impact of Ghana's free maternal healthcare policy under the NHIS. However, qualitative findings revealed important gaps between policy intent and lived experience. Women consistently reported out-of-pocket payments for essential items such as gloves and medicines, alongside high transportation costs, particularly during emergencies and in remote communities. These “hidden costs” undermine the promise of free maternal healthcare and have been widely documented in similar settings, where indirect and opportunity costs continue to limit access despite fee exemption policies (28, 29).

The quantitative results further revealed a nuanced relationship between transport costs and skilled delivery utilization. Although transport costs were significant in bivariate analysis, they did not remain independently associated in the multivariable model, suggesting that transport barriers are embedded within broader structural constraints such as income level, distance to facilities, and employment status. Qualitative narratives reinforced this interpretation, illustrating how cumulative costs, rather than transport expenses alone, effectively exclude poorer women living farther from health facilities. In such contexts, financial barriers function as composite constraints, where multiple disadvantages converge to restrict access to skilled care.

Beyond economic considerations, socio-cultural beliefs and household power dynamics played a substantial role in shaping delivery decisions. A notable proportion of women perceived childbirth as a natural process that does not necessarily require medical intervention, expressed a preference for home delivery or TBAs, and reported limited autonomy in deciding their place of delivery. Male dominance in reproductive health decision-making (AOR = 0.5) and a preference for TBAs (AOR = 0.6) independently reduced the likelihood of skilled delivery utilization. Qualitative findings provided critical context, revealing that trust in TBAs was rooted in cultural familiarity, emotional support, shared language, and perceived spiritual protection. Similar patterns have been reported in other LMIC settings, where TBAs are often viewed as more compassionate, accessible, and culturally aligned than formal health providers (30, 31). In addition, religious beliefs framing childbirth outcomes as determined by divine will sometimes diminished the perceived need for medical care. These findings suggest that increasing skilled delivery utilization requires culturally sensitive strategies that engage community belief systems, foster collaboration with traditional providers, and promote shared decision-making within households, particularly by involving male partners.

Health system factors further compounded these challenges and emerged as some of the strongest predictors of non-utilization. Geographical accessibility remained a major barrier, with women residing five kilometers or more from a health facility having significantly lower odds (AOR = 0.4) of skilled delivery use. Long travel times, poor road networks, and the absence of reliable transport, especially at night, exacerbated these difficulties. These findings mirror evidence from other rural and semi-urban LMIC contexts, where distance and transport constraints consistently predict lower rates of facility-based delivery (32, 33). However, physical access alone did not fully explain utilization patterns. Perceived quality of care, particularly staff attitudes, was a critical determinant. Negative experiences with healthcare workers, reflected in both quantitative associations (AOR = 0.4) and qualitative accounts of disrespectful or dismissive treatment, generated fear and mistrust, discouraging women from seeking facility-based delivery in subsequent pregnancies. This aligns with global evidence demonstrating that disrespectful and abusive maternity care undermines service utilization and erodes confidence in health systems (9, 34).

Importantly, the mixed-methods design enabled meaningful triangulation of findings. Quantitative associations, such as the strong influence of health insurance or cultural norms, were enriched by qualitative narratives that explained how these factors operate in practice through hidden costs, familial pressure, spiritual beliefs, and interpersonal experiences within health facilities. This integration confirms that barriers to skilled delivery are interrelated rather than isolated, operating synergistically to shape women's choices. Consequently, interventions aimed at improving skilled delivery utilization must address not only financial and infrastructural constraints but also socio-cultural norms, household power relations, and the quality of interpersonal care within health facilities.

### Strengths and limitations

A key strength of this study is its mixed-methods design, which provided both measurable associations and deep contextual understanding of the “why” behind the numbers. The community-based sampling and inclusion of diverse stakeholders (women, TBAs, health workers, community leaders) enhance the credibility and transferability of the findings. However, the study is not without limitations. Although causality cannot be inferred, the consistent associations and qualitative narratives suggest plausible pathways, such as economic empowerment enabling the overcoming of geographical barriers, or experiences of disrespect eroding trust irrespective of physical access. The reliance on self-reported data, particularly for sensitive topics such as household decision-making, may be subject to social desirability bias. Furthermore, the study was conducted in one municipality, which may limit the generalizability of findings to other regions with different socio-cultural or health system dynamics.

### Implications for policy and practice

The findings of this study offer several actionable insights for both local health planners and national policymakers seeking to improve skilled delivery service utilization. First, there is an urgent need to strengthen financial protection mechanisms within Ghana's Free Maternal Healthcare Policy by conducting regular audits and enforcing compliance to eliminate unofficial charges at health facilities. Although the policy aims to remove direct financial barriers, the persistence of out-of-pocket payments undermines its effectiveness. Complementary interventions, such as transportation vouchers, emergency transport subsidies, or community-based ambulance schemes, could further reduce the financial and logistical barriers associated with reaching health facilities, particularly for women in remote communities and during obstetric emergencies.

In addition to financial reforms, addressing socio-cultural barriers requires deliberate engagement with key community actors who influence maternal health decisions. Health promotion initiatives should be co-designed with communities, including TBAs and religious leaders, to ensure cultural relevance and local ownership. Rather than marginalizing TBAs, programs that provide training and formally integrate them as referral agents or community liaisons within the health system may help bridge cultural divides and promote timely facility-based delivery. Furthermore, targeted education and dialogue initiatives involving men are essential, given their dominant role in household decision-making. Promoting supportive spousal involvement can enhance women's autonomy and facilitate shared decision-making around place of delivery.

Improving the responsiveness and quality of the health system is equally critical. Strategic investments are needed to expand the coverage of primary healthcare facilities, particularly Community-based Health Planning and Services (CHPS) compounds, to reduce geographical barriers to skilled delivery. However, improvements in physical infrastructure must be accompanied by investments in “soft” infrastructure, including continuous professional development for healthcare providers focused on respectful maternity care, patient rights, and effective communication. Addressing staffing shortages and excessive workloads is also necessary, as these systemic pressures contribute to negative provider attitudes and compromise the quality of interpersonal care, ultimately deterring women from utilizing skilled delivery services.

Finally, the findings underscore the importance of adopting an intersectional approach to intervention design. The barriers identified in this study do not operate independently but intersect across economic, social, and health system domains. For instance, transportation subsidies alone may have limited impact if women lack decision-making autonomy within their households or fear mistreatment at health facilities. Effective programs must, therefore, be comprehensive and coordinated, addressing financial constraints, socio-cultural norms, and systemic health system weaknesses simultaneously to achieve sustained improvements in skilled delivery utilization.

## Conclusion

This study confirms that underutilization of skilled delivery services in West Akim Municipality is a multifaceted problem rooted in economic constraints, cultural preferences, and health system deficiencies. While financial policies like the NHIS are necessary, they alone are insufficient. Achieving significant improvements in skilled delivery coverage and, consequently, reductions in maternal mortality, requires a holistic strategy. This strategy must simultaneously enhance economic accessibility through robust financial protection, foster community trust through culturally sensitive engagement and quality improvement, and ensure geographical availability through prudent health system expansion. Future research should explore longitudinal and intervention-based designs to test the effectiveness of integrated approaches addressing these intertwined barriers.

## Data Availability

The original contributions presented in the study are included in the article/Supplementary Material, further inquiries can be directed to the corresponding author.
