# Supplementary material for: Factors affecting skilled delivery service utilization: a mixed-methods study in Ghana's West Akim Municipality in the Eastern Region
Source: Front Glob Womens Health. 2026 Jun 17;7:1795305. doi: 10.3389/fgwh.2026.1795305 (PMC13318948; doi:10.3389/fgwh.2026.1795305)
Supplement: Supplementary file 1 [file Table1.docx]

**Supplementary Material**

Supplementary Table 1**:** Standards for Reporting Qualitative Research (SRQR)

| Standards for Reporting Qualitative Research (SRQR) | | |
| --- | --- | --- |
| No. | Topic | Page (section) in manuscript |
|  | **Title and abstract** |  |
| S1 | Title | Page 1 (Title) |
| S2 | Abstract | Page 2 (Abstract) |
|  | **Introduction** |  |
| S3 | Problem formulation | Pages 3-4 (Introduction) |
| S4 | Purpose or research question | Page 2 (Introduction, final paragraph): "This study, therefore, aimed to..." |
|  | **Methods** |  |
| S5 | Qualitative approach and research paradigm | Page 2 (Methods: Study Design): Describes Convergent Parallel Mixed-Methods. Page 13 (Data Analysis): Describes Braun & Clarke thematic analysis. |
| S6 | Researcher characteristics and reflexivity | N/A |
| S7 | Context | Page 2 (Methods: Study Design and Setting): Describes Asamankese District, West Akim Municipality, and includes Figure 1 map. |
| S8 | Sampling strategy | Pages 2-3 (Study Population and Sampling): Describes purposive sampling for FGDs, IDIs, KIIs. |
| S9 | Ethical issues pertaining to human subjects | Page 3 (Ethical Considerations): IRB approval (Ref No. PUG/ST/AS/24070035), consent process, minor assent, confidentiality. |
| S10 | Data collection methods | Page 3 (Data Collection): Describes FGDs (n=28), IDIs (n=20), KIIs (n=16) and session durations. |
| S11 | Data collection instruments and technologies | Page 3 (Data Collection): Semi-structured guides, audio recording, Kobo Toolbox. |
| S12 | Units of study | Page 3 (Data Collection) & Page 4 (Results intro): 4 FGDs, 20 IDIs, 16 KIIs. |
| S13 | Data processing | Page 3 (Data Collection, paragraph 3): Describes two-step verification protocol (Twi transcription -> English translation -> 10% back-translation). |
| S14 | Data analysis | Page 3 (Data Analysis, paragraph 2): Braun & Clarke's six-phase framework cited. Inductive approach. |
| S15 | Techniques to enhance trustworthiness | Page 3 (Data Analysis, paragraph 2): Triangulation, peer debriefing, reflexive journaling. |
|  | **Results/findings** |  |
| S16 | Synthesis and interpretation | Pages 4-6 (Results): Findings structured by Theme with integrated quantitative tables and qualitative narratives. |
| S17 | Links to empirical data | Pages 4-6 (Results): Multiple quotes provided with specific identifiers (e.g., "FGD participant, rural, age 28", "KII, Midwife"). |
|  | **Discussion** |  |
| S18 | Integration with prior work, implications,  transferability, and contribution(s) to the field | Pages 6-7 (Discussion): Comparison with LMIC literature. |
| S19 | Limitations | Page7 (Strengths and Limitations): |
|  | **Other** |  |
| S20 | Conflicts of interest | Page 9 (Competing interests) |
| S21 | Funding | Page 9 (Funding): "This research received no specific grant..." |
|  |  |  |

**Supplementary Table 2:** Socio-demographic Characteristics of Survey Participants (N=402)

| **Characteristic** | **Category** | **Frequency (n)** | **Percentage (%)** |
| --- | --- | --- | --- |
| Age Group (years) | 15–24 | 118 | 29.4 |
|  | 25–34 | 196 | 48.8 |
|  | 35–49 | 88 | 21.9 |
| Marital Status | Single | 62 | 15.4 |
|  | Married/Cohabiting | 298 | 74.1 |
|  | Divorced/Widowed | 42 | 10.5 |
| Education Level | No formal education | 56 | 13.9 |
|  | Primary | 112 | 27.9 |
|  | Secondary | 162 | 40.3 |
|  | Tertiary | 72 | 17.9 |
| Parity | 1 | 96 | 23.9 |
|  | 2–3 | 204 | 50.7 |
|  | 4+ | 102 | 25.4 |
| Employment Status | Employed | 244 | 60.7 |
|  | Unemployed | 158 | 39.3 |

*Note: GHS: Ghana Cedis. Exchange rate at time of study (January 2025): 1 USD ≈ 15.0 GHS. Mean transport cost was approximately $0.83 USD (skilled group) vs $1.25 USD (non-skilled group). Household income categories were defined by sample tertiles: Low (<800 GHS/month), Medium (800-1500 GHS/month), High (>1500 GHS/month).*

**Supplementary Table 3:** Characteristics of Qualitative Study Participants

| Participant Type | Number | Key Characteristics |
| --- | --- | --- |
| FGD Members | 28 | - Postpartum women (within 12 months). |
| IDI Participants | 20 | - Skilled delivery users and non-users |
| KIIs | 16 | - Healthcare Workers: Midwives/Nurses from local facilities. - Traditional Birth Attendants: Practicing in study communities. - Religious Leaders: Pastors/Imams from local congregations. - Community Leaders: Assembly members/chiefs. |
